# The Brown Strain of *Flammulina velutipes* Singer Attenuates 5-Fluorouracil-Induced Intestinal Injury by Suppressing Inflammation, Oxidative Stress, and Barrier Disruption via Modulation of Epithelial–Mesenchymal Transition and Tight Junction Integrity

**DOI:** 10.3390/ijms27073212

**Published:** 2026-04-01

**Authors:** Sheng-Hsiung Huang, Hung-En Liao, Wen-Ping Jiang, Atsushi Inose, Wen-Liang Wu, Guan-Jhong Huang

**Affiliations:** 1 Department of Healthcare Administration, Asia University, Taichung 413, Taiwanheliao@asia.edu.tw (H.-E.L.); 2 School of Pharmacy, China Medical University, Taichung 404, Taiwan; wpjiang@cmu.edu.tw; 3 Faculty of Pharmacy, Nihon Pharmaceutical University, Saitama 362-0806, Japan; ainose@nichiyaku.ac.jp; 4 School of Chinese Medicine, College of Chinese Medicine, China Medical University, Taichung 404, Taiwan; 5 Department of Food Nutrition and Healthy Biotechnology, Asia University, Taichung 413, Taiwan; 6 Department of Chinese Pharmaceutical Sciences and Chinese Medicine Resources, College of Chinese Medicine, China Medical University, Taichung 404, Taiwan

**Keywords:** brown-strain *Flammulina velutipes*, 5-fluorouracil, mucositis, oxidative stress, inflammation, apoptosis, epithelial–mesenchymal transition, tight junction, PI3K/AKT axis

## Abstract

5-Fluorouracil (5-FU) remains a cornerstone chemotherapeutic for colorectal cancer, exerting its antitumor effects primarily through disruption of DNA and RNA synthesis and subsequent induction of apoptosis. Nonetheless, its clinical efficacy is often compromised by prominent adverse effects, particularly mucositis. This study examines the potential of brown-strain *Flammulina velutipes* Singer (FVB) to alleviate 5-FU-associated intestinal damage in a mouse model, offering insights into its possible role in mitigating chemotherapy-induced toxicity. 5-FU treatment significantly exacerbated gastrointestinal toxicity, as evidenced by severe diarrhea, shortened colon length, villus atrophy, and architectural disorganization of the intestine. It also inhibited crypt cell proliferation and induced body weight loss. Mechanistically, 5-FU activated pro-inflammatory, apoptotic, oxidative stress, and EMT pathways and disrupted mucosal tight junctions. Notably, FVB administration mitigated these pathological changes, indicating its protective role against 5-FU-induced intestinal injury. In summary, this investigation presents novel evidence for the protective role of FVB in mitigating 5-FU-induced intestinal mucositis. The results highlight the therapeutic potential of FVB as an adjunct to chemotherapy, potentially reducing treatment-related toxicity and enhancing the clinical care and quality of life of individuals undergoing colorectal cancer therapy.

## 1. Introduction

Chemotherapy, in combination with radiotherapy and surgery, is integral to modern cancer therapy. It has significantly enhanced long-term survival as a cornerstone treatment for systemic malignancies. However, its greatest limitation lies in the absence of tumor-specific targeting. Traditional chemotherapeutic agents such as paclitaxel and cisplatin indiscriminately attack rapidly dividing cells, often damaging healthy tissues and inducing systemic immune suppression [1]. The most affected sites include rapidly renewing tissues, i.e., the oral mucosa (painful ulceration), gastric mucosa (vomiting), and especially the intestinal epithelium, whose cells are replenished every 48 h and thus suffer severe injury. This toxicity highlights the urgent need for more targeted and protective approaches [2]. Patient susceptibility to chemotherapy-induced intestinal mucositis varies according to the type and dosage of chemotherapeutic agents. Approximately 50% to 80% of patients receiving certain regimens develop gastrointestinal complications, including mucosal bleeding, diarrhea, abdominal discomfort, malnutrition, and increased susceptibility to infection [3]. Intestinal mucositis primarily affects rapidly proliferating intestinal epithelial cells and is characterized by cellular infiltration, apoptosis, inflammatory responses, and disruption of epithelial barrier integrity. These pathological changes significantly impair patient quality of life and compromise the therapeutic efficacy of anticancer regimens [4]. In severe cases, mucositis may lead to treatment delays, dose reductions, or even discontinuation, ultimately reducing survival outcomes.

5-Fluorouracil (5-FU) is a fundamental chemotherapeutic agent in the management of colorectal, gastric, breast, and head and neck malignancies. As a classic antimetabolite, 5-FU primarily exerts its cytotoxic effects by inhibiting thymidylate synthase, thereby disrupting de novo thymidine synthesis, and by incorporation into DNA and RNA, leading to chain termination and inhibition of nucleic acid synthesis. This mechanism confers selective cytotoxicity against rapidly proliferating tumor cells and underpins its role in many established combination chemotherapy regimens [5]. However, it also adversely affects normal proliferative tissues, especially the gastrointestinal epithelium, often leading to intestinal mucositis, diarrhea, and immunosuppression. Current therapeutic approaches for mucositis are largely supportive, aiming to alleviate symptoms using mucosal protectants, analgesics, or antibiotics. Thus, there is an urgent need for novel adjuvant therapies that mitigate these toxicities while maintaining chemotherapeutic efficacy [6]. Therapeutic strategies for intestinal mucositis include mucosal protectants, immunomodulators, growth-promoting factors, and anti-inflammatory agents. These modalities collectively target mucosal integrity, immune regulation, tissue regeneration, and inflammation resolution, offering potential to mitigate the debilitating effects of chemotherapy-induced damage and improve outcomes in affected patients [7]. In recent years, complementary therapies—such as Chinese herbal medicine, dietary supplements, and enzymes—have shown increasing promise in addressing intestinal mucositis. While no single agent achieves complete efficacy, several interventions have proven beneficial in specific clinical scenarios. Notable examples include palifostine (a free radical scavenger), palifermin (a growth factor promoting mucosal repair), probiotics (e.g., Lactobacillus species), zinc sulfate, and antioxidants such as cysteine and L-glutamine. These options offer valuable adjunctive support in mitigating chemotherapy-induced mucosal damage [8].

Mucositis, one of the most common dose-limiting side effects of chemotherapy, develops through a series of well-defined, interconnected stages: initiation, primary injury response, amplification, ulceration, and eventual healing. The pathological cascade is triggered by DNA strand breaks and the generation of reactive oxygen species (ROS), which directly inflict damage upon the intestinal epithelial lining and the underlying submucosal tissues [9]. These injuries activate key signaling pathways, especially NF-κB, which mediates the expression of inflammatory cytokines and propagates tissue damage through a positive feedback loop. Ulceration follows, marked by mucosal disintegration and high infection risk. Healing involves epithelial migration and restoration of tissue structure [10]. The cytotoxic effects of chemotherapy, including inhibited DNA replication and increased oxidative stress, further exacerbate apoptosis and inflammation, highlighting the need for adjunct therapies that target these mechanisms [11].

The brown variant of *Flammulina velutipes* (FVB), commonly called golden Nameko or Jinhua mushroom, is a pigmented enoki strain with documented antioxidant properties. These bioactivities support its emerging role in functional foods, nutraceuticals, and adjunctive therapy [12]. Additionally, the FVB extract employed in this study was found to contain appreciable quantities of bioactive phenolic constituents. Quantitative high-performance liquid chromatography (HPLC) analysis revealed the presence of gallic acid and quercetin at concentrations of 8.83 μg/g and 66.93 μg/g, respectively. Both compounds are recognized for their chemoprotective properties, including anti-inflammatory and antioxidant activities, thereby providing a plausible mechanistic rationale for the protective effects observed in the present investigation [13]. Quercetin administration has been shown to significantly alleviate 5-FU-induced oral mucositis in mice [13], while gallic acid has been found to exert protective effects against doxorubicin- and 5-FU-induced hepatic and renal toxicity in rat models [14,15]. This study sought to determine whether FVB confers protective effects against 5-FU-induced intestinal mucositis and to delineate the mechanisms by which it exerts these effects. By clarifying how FVB intervenes in this pathological cascade, we aim to establish a theoretical rationale for its use as a dietary or therapeutic strategy to preserve intestinal integrity and reduce chemotherapy-related mucosal damage.

## 2. Results

### 2.1. The Effect of FVB on Clinical Indicators (Weight Loss, Diarrhea Severity, Intestinal Length) Was Systematically Assessed in the 5-FU-Induced Mucositis Model

**Figure 1:**
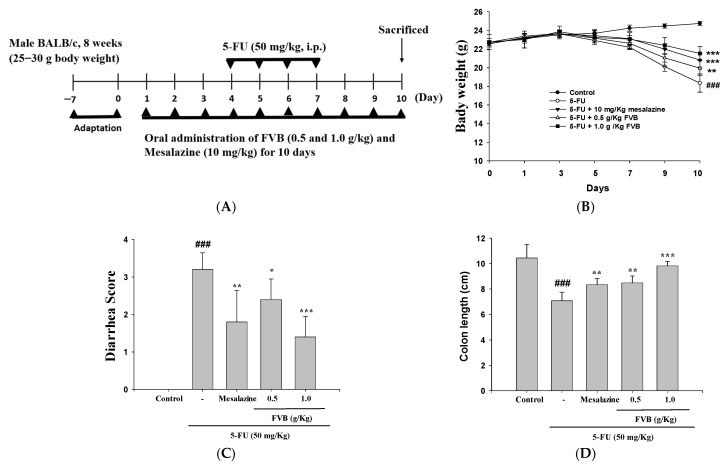
The ameliorative effect of FVB against 5-FU-induced intestinal mucositis in mice was confirmed through comprehensive physical and histological evaluations. As illustrated in the experimental scheme (**A**), key parameters, including body weight (**B**), diarrhea scores (**C**), and colon length (**D**), were systematically assessed. Following a 10-day administration regimen of FVB (0.5 or 1.0 g/kg/day), with 5-FU injected consecutively from day 4 to day 7, all mice were euthanized on day 10 for subsequent analysis. Data (mean ± S.D., n = 5) show significant differences: ^###^ *p* < 0.001 vs. control; * *p* < 0.05, ** *p* < 0.01, *** *p* < 0.001 vs. 5-FU group.

A systematic assessment was conducted to evaluate the impact of FVB on key clinical indicators (weight loss, diarrhea severity, and intestinal length) in the 5-FU-induced intestinal mucositis model (Figure 1A). Weight and fecal consistency were key parameters for assessing disease progression. Control group mice exhibited normal activity, healthy fur, adequate hydration, and no signs of gastrointestinal distress. In contrast, mice administered 5-FU (50 mg/kg) began to show clinical symptoms by day 5, including watery diarrhea, hypoactivity, and reduced food consumption. By day 8, symptoms progressed to severe diarrhea accompanied by hematochezia and postural abnormalities. Notably, body weight loss became statistically significant between days 4 and 7, correlating with the onset and progression of diarrhea. Compared to the gradual weight gain observed in the control group, mice administered 5-FU experienced notable body weight loss, consistent with the systemic toxicity associated with chemotherapy. However, prophylactic treatment with FVB led to significant, dose-dependent improvements in physical symptoms, most pronounced at doses of 0.5 g/kg and 1.0 g/kg (Figure 1B). These findings suggest a potential protective mechanism through which FVB mitigates 5-FU-induced intestinal mucositis, likely by attenuating epithelial damage and associated inflammatory responses. Quantification of diarrhea severity using the Bowen scoring system (ranging from 0 to 3) revealed a statistically significant reduction in symptom intensity among FVB-treated mice by day 10, in contrast to the untreated 5-FU group (Figure 1C). These findings strongly support the potential therapeutic role of FVB in preserving intestinal homeostasis and mitigating chemotherapy-induced mucosal damage.

To evaluate structural alterations associated with intestinal injury, colon length was measured as a histopathological indicator. Mice in the 5-FU group exhibited pronounced colon shortening, averaging 7.1 cm, significantly less than the 10.4 cm observed in the control group. Notably, FVB treatment at 1.0 g/kg mitigated this pathological feature, preserving colon length at 9.8 cm despite ongoing 5-FU administration (Figure 1D). These results suggest that FVB exerts protective effects on the intestinal architecture, likely through mechanisms involving anti-inflammatory and epithelial-regenerative pathways.

### 2.2. Protective Effects of FVB Against 5-FU-Induced Histopathological Damage in Intestinal Tissue

**Figure 2:**
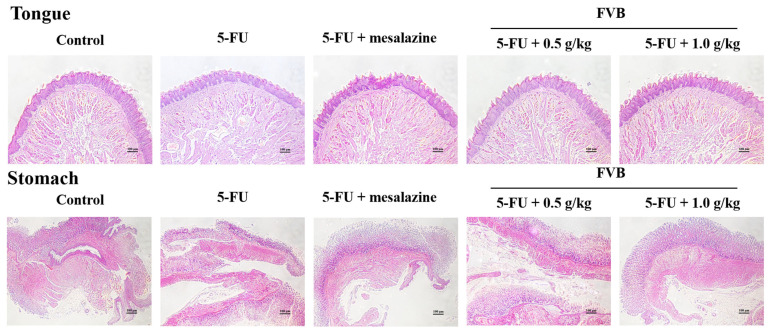

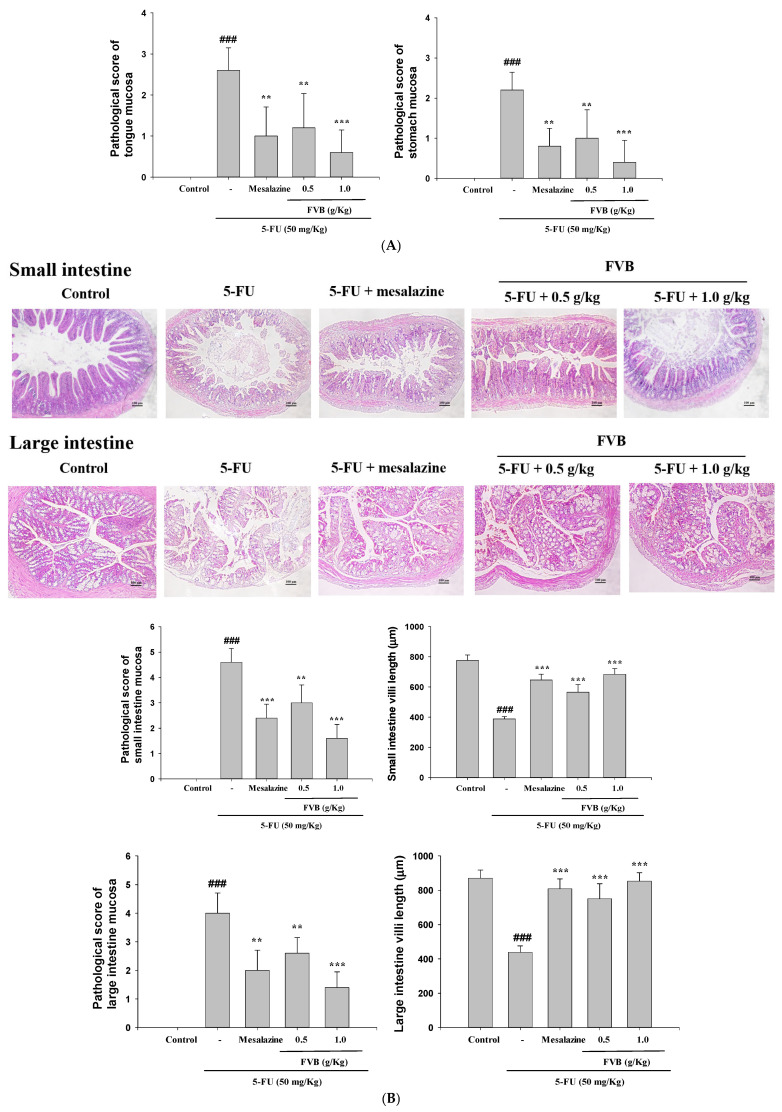

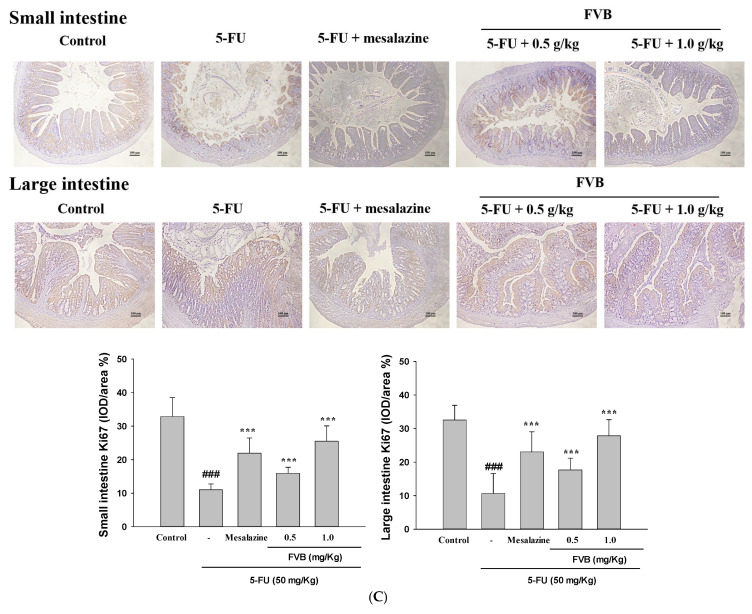
Histomorphological and immunohistochemical analyses were central to assessing the intestinal response to 5-FU with or without FVB cotreatment. Specifically, H&E staining was applied to tongue, stomach, small intestine, and colonic specimens (**A**,**B**), while Ki-67 immunolabeling was performed on small and large intestinal sections (**C**) to gauge crypt proliferative activity. The dosing schedule comprised 10 consecutive days of oral FVB (0.5 or 1.0 g/kg), with 5-FU administered intraperitoneally during days 4–7. On day 10, tissues were meticulously harvested and processed for high-resolution microscopic interrogation. Data (mean ± S.D., n = 5) demonstrated significant differences: ^###^ *p* < 0.001 vs. control; ** *p* < 0.01, *** *p* < 0.001 vs. 5-FU. Magnification, ×100; scale bar, 100 µm.

Histopathological examination of intestinal tissues using hematoxylin and eosin (H&E) staining revealed that 5-FU administration induced pronounced mucosal damage in the tongue, stomach, small intestine, and large intestine. As depicted in Figure 2A, the 5-FU-treated group exhibited substantial disruption of the tongue epithelial architecture, characterized by keratinized layer peeling and epithelial atrophy. In contrast, mice receiving FVB pretreatment demonstrated markedly attenuated histopathological alterations. These findings were further substantiated by quantitative histopathological scoring, thereby confirming the protective efficacy of FVB against 5-FU-induced mucosal injury (Figure 2A). In the control group, gastric sections showed normal surface epithelial cells, foveolar sections, glandular epithelial cells, submucosa, muscle layer, and serosa. In the mucositis group, surface epithelial cell degeneration, edema, and widespread glandular enlargement were observed. Epithelial cell degeneration manifested as focal changes. In the mucositis + FVB group, epithelial cell degeneration, edema, and glandular enlargement were less pronounced (Figure 2A). Control animals displayed intact villous architecture, characterized by tall columnar epithelium, numerous goblet cells, and regularly shaped crypts with active mitosis. By stark contrast, mice challenged with 5-FU exhibited severe intestinal injury: crypt cells were almost completely lost, villi were markedly shortened and blunted, epithelial vacuolization was widespread, and the submucosa appeared edematous with pronounced leukocytic infiltration into the lamina propria (Figure 2B). These structural damages are consistent with clinical presentations of intestinal mucositis, reflecting both the cytotoxic and inflammatory consequences of 5-FU. Notably, pretreatment with FVB markedly alleviated these histological disruptions. Mice receiving FVB showed preserved villus height and morphology, reduced apoptosis of crypt epithelial cells, and a significant decrease in inflammatory cell infiltration. Additionally, the intestinal architecture in FVB-treated animals more closely resembled that of the control group, suggesting enhanced mucosal integrity and regenerative capacity. These improvements were more pronounced at the 1.0 g/kg dose, indicating a potential dose-dependent protective effect.

Villus height is a sensitive morphometric parameter reflecting the extent of mucosal injury. In the present study, histological evaluation demonstrated elongated and orderly villi in the control group, while 5-FU injection produced severe mucosal disruption with a marked decrease in villus height. Remarkably, mice receiving FVB alongside 5-FU retained significantly taller villi than those treated with 5-FU alone (Figure 2B), suggesting that FVB affords notable protection to the intestinal epithelium. Taken together, these histological findings underscore the therapeutic potential of FVB in ameliorating 5-FU-induced intestinal injury. The preservation of mucosal structure and suppression of inflammatory damage suggest that FVB not only mitigates epithelial destruction but may also support epithelial regeneration and the restoration of barrier function. This highlights the clinical relevance of FVB as a complementary strategy for managing chemotherapy-induced mucositis during cancer treatment.

### 2.3. Ki-67 Immunoreactivity Was Markedly Increased in the FVB-Treated Group, Indicating a Restoration of Cellular Proliferative Activity in the Mucositis Model

Immunohistochemical analysis using Ki-67 staining demonstrated robust proliferative activity confined to the crypt region in the control group, reflecting normal epithelial turnover and homeostasis. In contrast, a significant decline in Ki-67 positivity was observed in mice administered 5-FU alone (*p* < 0.01), consistent with the known cytotoxic impact of 5-FU on rapidly dividing cells in the intestinal epithelium. Notably, FVB administration effectively restored Ki-67 expression to levels comparable to those of the control group, indicating a reversal of the antiproliferative effects of 5-FU (Figure 2C). This recovery of proliferation capacity may contribute to mucosal regeneration and the maintenance of epithelial barrier function, both of which are critical in limiting the progression of mucositis and preventing secondary infections or systemic inflammation. The small intestine was selected for this analysis due to its high cell turnover rate and its susceptibility to chemotherapeutic injury, as well as its relatively greater tissue availability compared to other gastrointestinal regions. These findings reinforce the protective and regenerative potential of FVB and provide mechanistic insight into its capacity to support intestinal recovery during chemotherapeutic insult.

### 2.4. FVB Administration Effectively Suppressed the Expression of Pro-Inflammatory Cytokines Induced by 5-FU

**Figure 3:**
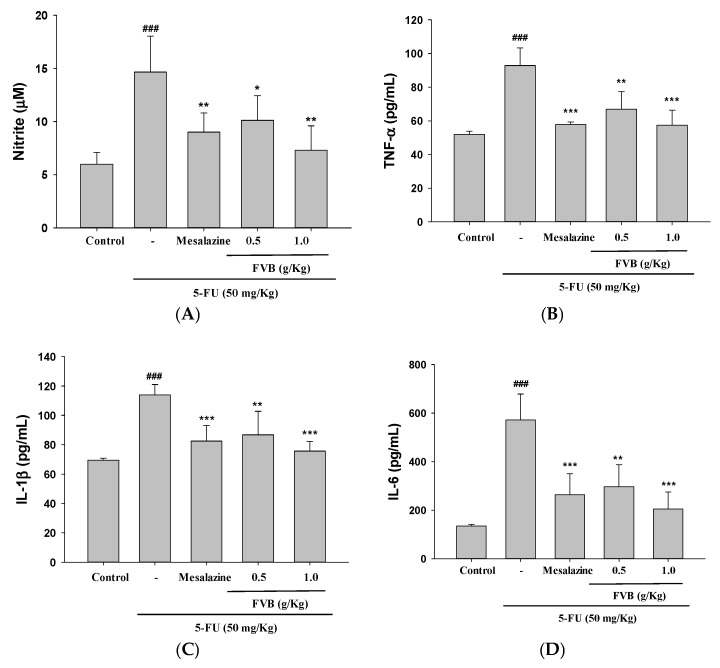
FVB exhibited anti-inflammatory properties in the 5-FU-induced intestinal mucositis model, as indicated by substantial reductions in serum concentrations of NO (**A**), TNF-α (**B**), IL-1β (**C**), and IL-6 (**D**). FVB was administered orally at 250 mg/kg and 500 mg/kg daily for 10 consecutive days, with 5-FU delivered intraperitoneally on days 4 to 7. Mice were sacrificed on day 10 for endpoint assessments. Data (mean ± S.D., n = 5) demonstrated significant differences: ^###^ *p* < 0.001 vs. control; *** *p* < 0.001, ** *p* < 0.01, and * *p* < 0.05, vs. 5-FU.

The mechanisms responsible for FVB’s protective effects against mucositis were investigated by analyzing inflammatory mediator expression in a 5-FU-induced murine model, with and without FVB administration. Since inflammation is a central pathophysiological feature of mucositis, pro-inflammatory cytokines serve as both diagnostic biomarkers and viable therapeutic targets. Figure 3A–D demonstrates that 5-FU administration led to substantial increases in NO, TNF-α, IL-1β, and IL-6 levels. Treatment with FVB (0.5 and 1.0 g/kg) markedly suppressed these elevations, restoring cytokine concentrations close to control values. Mesalazine (10 mg/kg), included as a positive control, exhibited a similar anti-inflammatory effect. These findings suggest FVB’s potential as an effective modulator of chemotherapy-induced inflammatory responses. These results indicate that FVB effectively suppresses key inflammatory mediators involved in 5-FU-induced mucosal injury. The cytokine-suppressive effects, likely attributed to the bioactive phytochemicals in FVB, point to a mechanistic role involving the modulation of oxidative stress and inflammatory signaling pathways.

### 2.5. FVB Alleviated Oxidative Stress in a 5-FU-Induced Intestinal Mucositis Model

**Figure 4:**
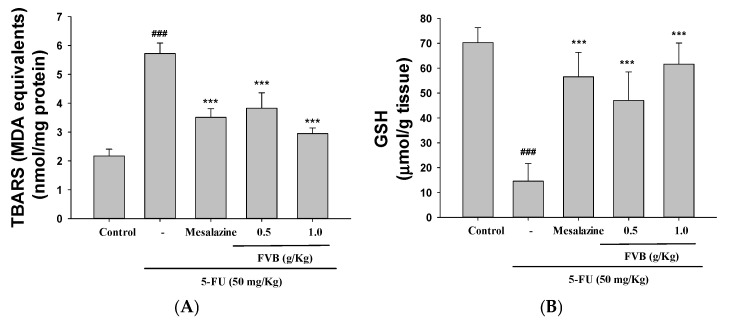
FVB effectively attenuated 5-FU-induced oxidative stress in the intestinal mucosa, as evidenced by TBARS (MDA equivalents) levels (**A**) and restored GSH content (**B**). Data (mean ± S.D., n = 5) demonstrated significant differences: ^###^ *p* < 0.001 vs. control; *** *p* < 0.001 vs. 5-FU.

Oxidative stress, a key contributor to mucosal injury in mucositis, was significantly attenuated by FVB administration. Analysis of lipid peroxidation and antioxidant markers revealed that 5-FU treatment markedly increased malondialdehyde (MDA) levels and reduced glutathione (GSH) content in the small intestinal mucosa. Pretreatment with FVB at 0.5 and 1.0 g/kg significantly suppressed MDA elevation and restored GSH levels toward baseline (Figure 4A,B), indicating FVB’s potential to modulate oxidative homeostasis under chemotherapeutic insult.

### 2.6. FVB Suppressed NF-κB and MAPK Pathway Activation and Reduced Inflammation in 5-FU-Induced Intestinal Mucositis

**Figure 5:**
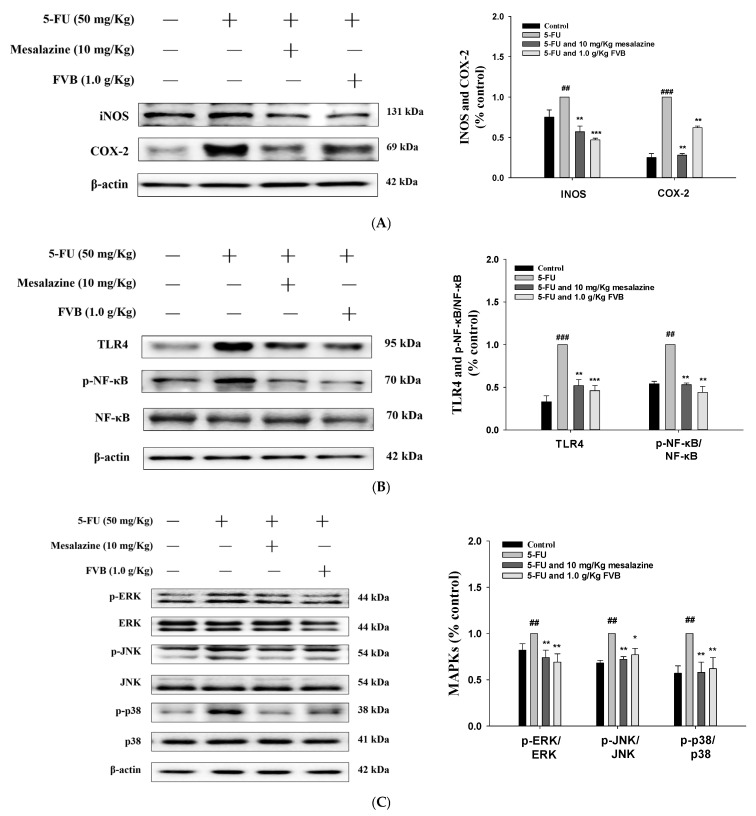
FVB treatment significantly downregulated the expression of iNOS and COX-2 (**A**), TLR4, IKK, phosphorylated IKK, NF-κB, phosphorylated NF-κB (**B**), and phosphorylated MAPK proteins (**C**) in the 5-FU-induced intestinal mucositis mouse model. Protein levels in the small intestine were determined via Western blot analysis and subsequent densitometric quantification. Data are presented as mean ± S.D. derived from at least three independent biological experiments, with protein expression levels normalized to β-actin as the loading control. Statistical significance is indicated as follows: ^##^ *p* < 0.01 and ^###^ *p*< 0.001 versus the control group; *** *p* < 0.001, ** *p* < 0.01, and * *p* < 0.05 versus the 5-FU-treated group. “+”: FVB or mesalazine treatment group; “−”: untreated control group.

In a murine model of 5-FU-induced intestinal mucositis, FVB treatment significantly mitigated inflammation and suppressed the activation of the NF-κB and MAPK signaling pathways. Moreover, the protein expression levels of cyclooxygenase-2 (COX-2) and inducible nitric oxide synthase (iNOS) were markedly downregulated following administration of FVB at 1.0 g/kg, an effect comparable to that observed with the anti-inflammatory control agent mesalazine. (Figure 5A). These findings demonstrate that FVB can effectively disrupt inflammatory cascades relevant to mucosal injury.

As innate immune receptors, toll-like receptors (TLRs)—particularly toll-like receptor 4 (TLR4)—mediate inflammation in response to cellular stressors such as chemotherapeutic agents. Western blot analysis showed robust TLR4 activation in the 5-FU group, whereas FVB pretreatment significantly inhibited this response (Figure 5B). This downregulation of TLR4 by FVB supports a mechanistic framework in which FVB interferes with upstream inflammatory signaling cascades, potentially contributing to its therapeutic efficacy in mucositis. The nuclear factor-κB (NF-κB) signaling pathway, which is essential for initiating inflammatory responses, has been implicated in numerous inflammatory conditions. In this study, elevated expression of phosphorylated NF-κB was detected in 5-FU-treated small intestinal tissues. These elevations were significantly suppressed following FVB pretreatment, suggesting that FVB exerts anti-inflammatory effects by targeting the TLR4/NF-κB signaling cascade (Figure 5B).

Given the established role of MAPK signaling in orchestrating inflammatory cascades, we investigated its status in the 5-FU-induced mucositis model. 5-FU exposure led to a pronounced increase in the phosphorylated forms of JNK, ERK, and p38, with unequivocal evidence of pathway engagement. Intervention with either FVB or mesalazine effectively disrupted this activation signature, suppressing phosphorylation to near-baseline levels. Importantly, total kinase expression remained invariant throughout, localizing the regulatory effect to post-translational modification rather than protein turnover (Figure 5C). These results suggest that FVB inhibits MAPK activation, thereby contributing to its anti-inflammatory effects.

### 2.7. FVB Restored Antioxidant Defenses and Stimulated HO-1/Nrf2 Activation in 5-FU-Induced Intestinal Mucositis

**Figure 6:**
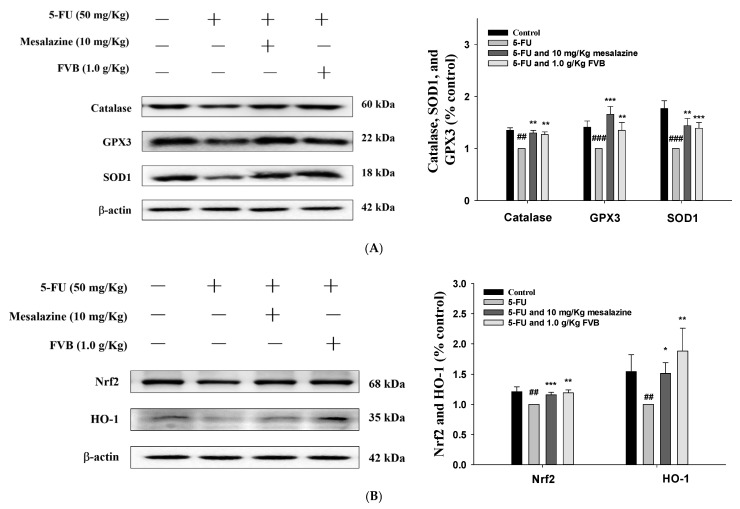
FVB treatment ameliorated the 5-FU-induced perturbations in the expression of antioxidant-related proteins. Specifically, it modulated the levels of catalase, SOD1, GPx3 (**A**), and components of the Keap1/Nrf2/HO-1 signaling axis (**B**) in small intestinal tissue. Protein levels in the small intestine were determined via Western blot analysis and subsequent densitometric quantification. Data are presented as mean ± S.D. derived from at least three independent biological experiments, with protein expression levels normalized to β-actin as the loading control. Statistical significance is indicated as follows: ^##^ *p* < 0.01 and ^###^ *p*< 0.001 versus the control group; *** *p* < 0.001, ** *p* < 0.01, and * *p* < 0.05 versus the 5-FU-treated group. “+”: FVB or mesalazine treatment group; “−”: untreated control group.

Oxidative stress is known to impair intestinal function and initiate inflammatory cascades. In this study, 5-FU administration led to decreased expression of catalase, SOD1, and GPx3 in intestinal tissues (Figure 6A). These reductions were largely restored by FVB pretreatment. Additionally, 5-FU exposure was associated with increased Keap1 and reduced Nrf2 and HO-1 expression (Figure 6B). These molecular alterations were attenuated by FVB, indicating its role in restoring redox balance during chemotherapy-induced mucositis.

### 2.8. FVB Attenuates the Activation of Apoptosis- and Autophagy-Related Signaling Pathways Triggered by 5-FU

**Figure 7:**
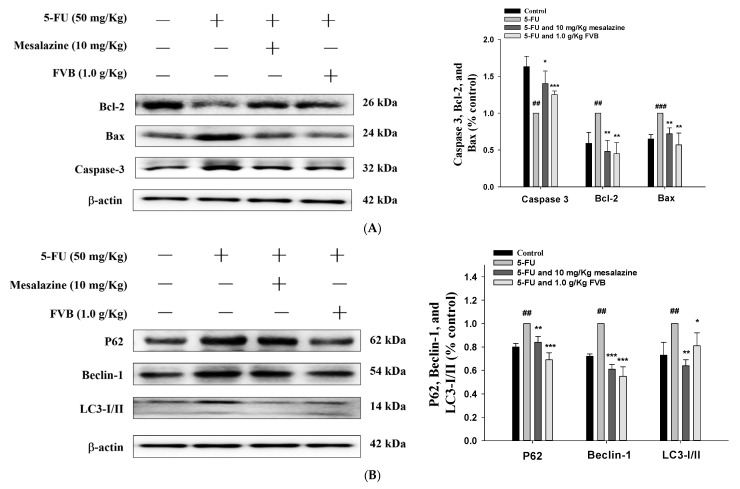
The modulatory effect of FVB on 5-FU-triggered apoptotic (**A**) and autophagic (**B**) cascades within the intestinal epithelium was investigated using quantitative immunoblotting. Targeted profiling of Bax, Bcl-2, caspase-3, p62, Beclin-1, and LC3-I/II was achieved using isoform-specific antibodies, with β-actin serving as a robust loading normalizer. Immunoreactive signals were captured, subjected to densitometric analysis, and ultimately normalized to β-actin. Data represent mean ± S.D. derived from a minimum of three biologically independent experiments, each performed with technical replicates. Statistical significance is indicated as follows: ^##^ *p* < 0.01 and ^###^ *p* < 0.001 versus the control group; *** *p* < 0.001, ** *p* < 0.01, and * *p* < 0.05 versus the 5-FU-treated group. “+”: FVB or mesalazine treatment group; “−”: untreated control group.

Immunoblotting analysis was performed to quantify the protein expression levels of apoptosis- and autophagy-related markers in intestinal tissue lysates obtained from the 5-FU-induced mucositis model, both in the presence and absence of FVB pretreatment. In the 5-FU monotherapy group, marked upregulation of Bax, Bcl-2, and caspase-3 was observed, indicative of apoptotic pathway activation. Conversely, these alterations were substantially reversed by FVB pretreatment, with protein levels restored toward baseline. These findings demonstrate that FVB exerts anti-apoptotic activity, thereby conferring protection against 5-FU-induced intestinal injury (Figure 7A). Parallel immunoblotting was performed to quantify autophagy-related markers, including p62, Beclin-1, and the LC3-II/I ratio. Significant increases in these parameters were detected in the 5-FU-treated group, reflecting impaired autophagic flux and autophagosome accumulation. Notably, FVB pretreatment markedly attenuated these changes, normalizing autophagy marker expression. Collectively, these results provide evidence that FVB modulates autophagic dysregulation, contributing to its protective effects in chemotherapy-induced mucositis (Figure 7B).

### 2.9. FVB Attenuates 5-FU-Induced Alterations in the PI3K/AKT Signaling Pathway

**Figure 8:**
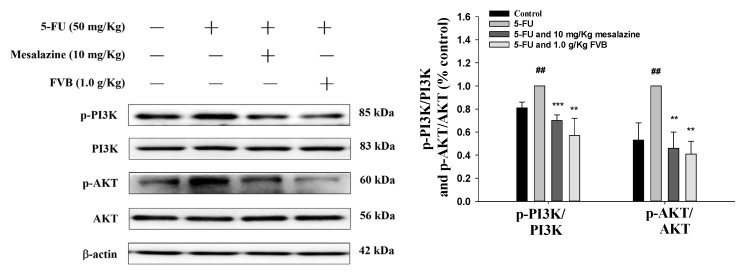
The protective effect of FVB against 5-FU-induced intestinal injury was associated with the attenuation of PI3K–Akt signaling axis activation. This modulatory action was evaluated by Western blot analysis of PI3K, phosphorylated PI3K (p-PI3K), Akt, and phosphorylated Akt (p-Akt) protein levels in small intestinal tissue lysates. Following immunoblotting, band intensities were quantified by densitometry, and all target protein expression levels were normalized to β-actin, which served as the internal loading control. Data are expressed as mean ± standard deviation (S.D.) derived from at least three independent biological experiments. Statistical significance is indicated as follows: ^##^ *p* < 0.01 versus the control group; *** *p* < 0.001, and ** *p* < 0.01 versus the 5-FU-treated group. “+”: FVB or mesalazine treatment group; “−”: untreated control group.

As a nodal point integrating proliferative cues and oxidative defense, the phosphatidylinositol-3-kinase (PI3K)/Akt (Protein kinase B, PKB) axis stands at the epicenter of mucositis pathophysiology. In the 5-FU-challenged intestine, this signaling hub became hyperactivated, manifesting as sharply elevated PI3K and Akt protein levels relative to basal conditions (Figure 8). FVB intervention, however, potently quenched this aberrant signal—effectively restoring pathway activity toward physiological setpoints. These findings indelibly implicate PI3K/Akt engagement as a critical conduit through which FVB exerts its mucosal cytoprotection.

### 2.10. The Epithelial–Mesenchymal Transition (EMT) Pathways Induced by 5-FU Were Alleviated by FVB Treatment

**Figure 9:**
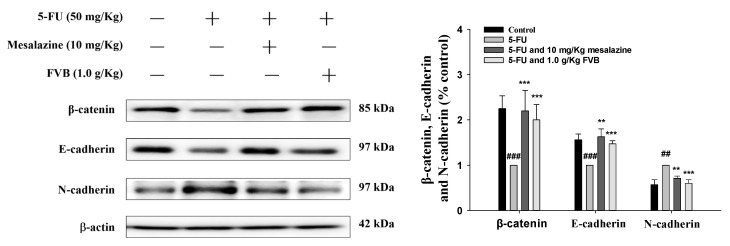
FVB intervention profoundly reshaped the EMT-associated protein landscape within the 5-FU-compromised intestinal epithelium. Quantitative immunoblotting, targeting β-catenin, E-cadherin, and N-cadherin, was deployed to capture this molecular reprogramming. Densitometric signals, rigorously normalized to the housekeeping protein β-actin, furnished robust quantitative readouts. All data represent mean ± S.D. derived from no fewer than three independent biological experiments. Statistical significance is indicated as follows: ^##^ *p* < 0.01 and ^###^ *p* < 0.001 versus the control group; *** *p* < 0.001, and ** *p* < 0.01 versus the 5-FU-treated group. “+”: FVB or mesalazine treatment group; “−”: untreated control group.

EMT has been implicated as a critical pathogenic event in 5-FU-induced intestinal mucositis, contributing to the extensive disruption of gastrointestinal epithelial integrity. EMT is characterized by the loss of epithelial hallmarks—most notably cell adhesion molecules—and the concurrent acquisition of mesenchymal phenotypic traits. As illustrated in Figure 9, exposure to 5-FU alone resulted in a significant downregulation of the epithelial markers β-catenin and E-cadherin, accompanied by marked upregulation of the mesenchymal marker N-cadherin. These molecular alterations were effectively reversed by FVB pretreatment, which restored β-catenin and E-cadherin expression while suppressing N-cadherin levels. Collectively, these findings indicate that FVB attenuates EMT-mediated epithelial damage, thereby revealing a putative therapeutic mechanism for the amelioration of 5-FU-associated mucositis.

### 2.11. FVB Alleviates the Disruption of Tight Junction Protein Expression Induced by 5-FU

**Figure 10:**
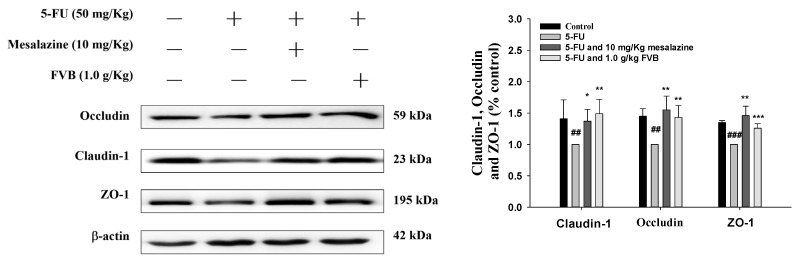
The expression of tight junction proteins—including ZO-1, occludin, and claudin—was significantly downregulated, thereby compromising epithelial barrier integrity in the 5-FU-induced intestinal mucositis model. FVB pretreatment effectively restored the levels of these proteins, suggesting a protective effect on barrier function. Protein expression was assessed by Western blotting using specific antibodies against ZO-1, occludin, and claudin, followed by densitometric quantification. All target protein levels were normalized to β-actin, which served as the internal loading control. Data are presented as mean ± S.D. derived from at least three independent biological experiments. Statistical significance is indicated as follows: ^##^ *p* < 0.01 and ^###^ *p*< 0.001 versus the control group; *** *p* < 0.001, ** *p* < 0.01, and * *p* < 0.05 versus the 5-FU-treated group. “+”: FVB or mesalazine treatment group; “−”: untreated control group.

The integrity of the intestinal epithelial barrier—a casualty of 5-FU-induced mucositis—hinges critically on the functional integrity of tight junction complexes, including ZO-1, occludin, and claudin-1. 5-FU administration precipitated a sharp, statistically significant erosion of these barrier guardians (Figure 10). FVB pretreatment, however, decisively reinstated their expression, effectively resealing the paracellular route and restoring mucosal homeostasis. These findings indelibly support the potential of FVB as a barrier-fortifying nutraceutical candidate, poised to mitigate chemotherapy-driven intestinal compromise.

## 3. Discussion

5-FU, a classic antimetabolite chemotherapeutic widely applied in colorectal, gastric, breast, and head and neck malignancies, primarily inhibits thymidylate synthase, leading to interference with DNA and RNA synthesis and subsequent cytotoxicity in rapidly proliferating cells. Despite its potent antitumor activity, its indiscriminate impact on normal proliferative tissues results in a broad range of dose-limiting toxicities that constrain its clinical application and compromise patient quality of life [16]. One of the most common and severe complications is gastrointestinal toxicity, characterized by inflammation and ulceration of the mucosal lining, which leads to abdominal cramping, diarrhea, nausea, and malabsorption. This not only impairs nutrient uptake but also exacerbates treatment-associated fatigue and weight loss. Additionally, bone marrow suppression induced by 5-FU compromises hematopoiesis, often resulting in leukopenia, thrombocytopenia, and anemia [17]. These hematologic effects increase susceptibility to infections and bleeding, while contributing to chronic fatigue and reduced treatment tolerance. These cumulative side effects often necessitate dose reductions or treatment discontinuation, underscoring the urgent need for supportive agents that can mitigate the 5 toxicities induced by 5-FU without interfering with its antitumor efficacy [18]. The selection of mesalazine as a positive control in this study was based on its well-established anti-inflammatory mechanisms, which directly target the key pathological pathways involved in 5-FU-induced intestinal mucositis. Mesalazine (5-aminosalicylic acid) is known to inhibit the production of pro-inflammatory cytokines such as TNF-α, IL-1β, and IL-6; suppress NF-κB activation; and reduce oxidative stress through reactive oxygen species scavenging [8]. These mechanisms align closely with the inflammatory cascade triggered by 5-FU in the intestinal mucosa, including immune cell infiltration, upregulation of COX-2 and iNOS, and disruption of epithelial barrier integrity [19]. Therefore, mesalazine represents a mechanistically appropriate and clinically relevant comparator for evaluating the anti-inflammatory efficacy of FVB in this model.

A critical consideration when interpreting in vitro data is the intrinsic nature of the cell models employed. Caco-2 cells, despite their ability to undergo enterocytic differentiation, are derived from a human colorectal carcinoma and therefore exhibit neoplastic characteristics, including altered metabolic enzyme expression and dysregulated signaling pathways. Given that 5-FU is a chemotherapeutic agent designed to target rapidly dividing malignant cells, its effects on Caco-2 monolayers may be disproportionately severe compared to those on healthy intestinal epithelium. This fundamental discrepancy raises concerns about the translational relevance of cytotoxicity data obtained from this cell line [20]. YAMC cells, while representing a non-transformed colonic epithelial population, are rendered immortal via transgenic expression of SV40 large T antigen. This genetic modification may perturb cell cycle checkpoints and DNA damage responses, potentially confounding assessments of drug-induced injury. Furthermore, both Caco-2 and YAMC monolayer systems are devoid of the multifaceted cellular interactions—involving immune cells, commensal microbiota, and mesenchymal support—that characterize the in vivo intestinal environment [21]. As such, they are inherently limited in their capacity to model the full spectrum of 5-FU-induced mucositis. A comprehensive understanding of 5-FU-induced intestinal mucositis necessitates consideration of its multifactorial etiology, which encompasses disruption of the mucous barrier, recruitment and activation of immune effector cells (e.g., macrophages and neutrophils), and alterations in the composition of the commensal microbiota [8]. Reductionist in vitro systems employing simple epithelial monolayers are fundamentally inadequate for modeling such complex intercellular and host–microbe interactions. Moreover, the fidelity of in vitro models is further compromised by discrepancies in drug metabolism. The activation and catabolism of 5-FU in vivo are governed by specific enzymes, including dihydropyrimidine dehydrogenase (DPD) and orotate phosphoribosyltransferase (OPRT). Caco-2 cells, owing to their neoplastic origin, exhibit an aberrant enzyme profile that may not faithfully replicate the metabolic fate of 5-FU in healthy tissue, thereby skewing assessments of cytoprotective interventions [22]. The absence of the mucus layer in conventional culture systems introduces an additional layer of complexity. Should the protective mechanism of a candidate compound involve mucus penetration or interaction, the absence of this physiological barrier in vitro could lead to over- or underestimation of its true bioavailability and therapeutic potential in vivo.

The severity of tissue injury induced by 5-FU in murine models is strongly dose-dependent, with higher doses correlating with more extensive pathological outcomes. Administration of high-dose 5-FU (e.g., 150 mg/kg or greater) typically leads to pronounced intestinal mucosal damage, systemic toxicity, and marked bone marrow suppression. These effects are commonly manifested as significant body weight loss, anorexia, diarrhea, and hematological abnormalities [23]. Such a model is often employed to simulate acute mucosal injury and to explore the pathophysiological mechanisms underlying severe chemotherapy-induced side effects. In contrast, lower doses of 5-FU (typically ≤50 mg/kg) induce relatively milder yet clinically relevant symptoms, including intestinal mucositis, without eliciting overt systemic toxicity. These low-dose models are particularly valuable for studying chronic or subacute mucosal injury, mimicking the prolonged clinical exposure patients often experience during repeated chemotherapy cycles. To investigate the dose–response relationship and assess the efficacy of potential therapeutic interventions, researchers commonly adjust the dosage of 5-FU based on the experimental objectives [24]. The low-dose 5-FU model is ideal for evaluating agents aimed at preventing or mitigating long-term mucosal damage with minimal systemic burden. Conversely, the high-dose model is more suitable for screening compounds with potent protective or regenerative effects under severe inflammatory and cytotoxic conditions. This dose stratification approach provides a flexible and controlled platform for examining not only the mechanistic basis of chemotherapy-induced mucosal injury but also the potential of candidate agents, such as anti-inflammatory, antioxidant, or mucosal barrier-protective compounds, to counteract or reverse 5-FU-induced toxicity. The ability to tailor 5-FU dosage in preclinical models thus plays a pivotal role in the development of targeted strategies to alleviate treatment-related complications in cancer patients [25]. In our investigation, mice dosed with 50 mg/kg 5-FU developed characteristic intestinal mucositis symptoms, including weight loss and diarrhea, with no mortality recorded. Notably, FVB treatment substantially reduced diarrhea severity, reinforcing its therapeutic promise and supporting its potential role in alleviating chemotherapy-induced intestinal damage in clinical settings.

5-FU dosage during oncologic treatment is precisely individualized according to multiple clinical variables, including tumor type and stage, patient age, and renal and hepatic function. A major dose-limiting toxicity of 5-FU is gastrointestinal mucositis, characterized by inflammation and ulceration throughout the gastrointestinal mucosa. Clinically, this presents as abdominal pain, diarrhea, malabsorption, gastrointestinal hemorrhage, and mucosal ulceration [26]. Among these, diarrhea is particularly detrimental, as it can lead to dehydration, electrolyte imbalances, malnutrition, and an increased risk of systemic infections. These complications often necessitate chemotherapy dose reductions, treatment delays, or complete discontinuation, which may compromise therapeutic efficacy [27]. Consequently, patients may experience prolonged treatment durations and higher healthcare costs due to extended hospital stays and the need for supportive care. Furthermore, chemotherapy-induced mucositis frequently results in decreased oral intake and impaired nutrient absorption, contributing to anorexia, significant weight loss, and a decline in overall patient performance status. These factors collectively diminish quality of life and may adversely affect treatment outcomes, underscoring the urgent need for effective interventions to mitigate intestinal toxicity while preserving the antitumor activity of 5-FU [28]. Chemotherapy-associated diarrhea has been reported to affect approximately 50–80% of patients, frequently emerging as a dose-limiting toxicity that compromises treatment continuity and diminishes quality of life. In the present murine model of intestinal mucositis, administration of 5-fluorouracil (5-FU) induced pronounced gastrointestinal injury, characterized by apoptosis of villous stem cells and suppression of epithelial proliferation, culminating in villous shortening and crypt atrophy. A progressive decline in body weight was evident by day 5 of 5-FU treatment, likely attributable to systemic toxicity, malabsorption, and fluid loss secondary to diarrhea. In contrast, mice co-treated with FVB exhibited a gradual recovery in body weight from day 5 to day 10, suggesting a protective effect against 5-FU-induced gastrointestinal toxicity. Notably, whereas diarrhea was consistently observed in the 5-FU monotherapy group, this symptom was completely absent in animals receiving FVB at 1.0 g/kg, indicating that FVB effectively mitigates chemotherapy-induced diarrhea [29]. These observations suggest that FVB has a therapeutic role in alleviating mucositis, as evidenced by improvements in weight maintenance, reduced diarrhea severity, and preservation of intestinal architecture. In this study, intestinal mucositis was induced in mice by intraperitoneal injection of 5-FU (50 mg/kg), followed by daily oral administration of FVB until day 10. Histopathological evaluation using H&E staining revealed that 5-FU-treated mice displayed significant pathological alterations, including villous blunting, crypt destruction, and increased infiltration of inflammatory cells within the mucosal layer [30]. Moreover, immunohistochemical staining for Ki-67, a marker of cellular proliferation, demonstrated a significant decline in proliferative activity in the 5-FU group, indicative of impaired mucosal regeneration. In contrast, FVB co-treatment preserved Ki-67 expression levels, supporting its role in enhancing intestinal repair processes and counteracting mucosal injury. The present findings collectively demonstrate that FVB confers significant protection against 5-FU-induced intestinal mucositis by enhancing mucosal integrity, attenuating inflammation, restoring proliferative capacity in the epithelium, and ameliorating gastrointestinal toxicities, including diarrhea and body weight loss. These results support the potential utility of FVB in mitigating chemotherapy-associated mucosal injury through modulation of inflammatory and regenerative pathways.

Cytokines derived from the intestinal immune system play a central role in safeguarding gut homeostasis—they orchestrate immune reactivity, fortify the epithelial barrier, and sustain a balanced microbiota. The delicate counterplay between pro- and anti-inflammatory cytokines is crucial: it enables protective immunity while restraining inflammatory overshoot that would otherwise inflict mucosal injury [31]. When this equilibrium collapses, it paves the way for gastrointestinal pathologies, including mucositis triggered by chemotherapeutic agents. In our experiments, 5-FU severely destabilized this cytokine network, driving pronounced overexpression of TNF-α, IL-1β, and IL-6 [32]. These molecules act as principal amplifiers of the inflammatory response and are robustly induced by cellular damage and oxidative stress [28]. However, pretreatment with FVB markedly suppressed the expression of TNF-α, IL-1β, and IL-6, indicating its effectiveness in re-establishing immunological balance and attenuating mucosal inflammation. This suggests that FVB may act as an immunomodulatory agent capable of mitigating chemotherapy-induced intestinal injury by restoring cytokine equilibrium.

Cancer therapies, particularly chemotherapy and radiation, often induce mucosal damage through two primary mechanisms: excessive generation of pro-inflammatory cytokines and the accumulation of ROS. 5-FU, a widely used chemotherapeutic agent, is known to produce ROS as part of its cytotoxic action against rapidly dividing tumor cells [33]. However, this ROS overproduction also damages healthy intestinal epithelial cells by oxidizing lipids, proteins, and DNA, thereby compromising cellular viability and intestinal barrier function. As epithelial cells become injured, they trigger the release of further inflammatory mediators—including TNF-α, IL-1β, and IL-6—from various sources such as immune cells, endothelial cells, and epithelial cells themselves. This amplifies the inflammatory cycle and contributes to the progression of mucositis [34]. The observed ability of FVB to suppress these key inflammatory cytokines suggests that it can interrupt this feedback loop of inflammation and oxidative stress, ultimately preserving intestinal integrity and function during 5-FU chemotherapy. Collectively, these findings underscore the therapeutic potential of FVB in counteracting the inflammatory and oxidative components of intestinal mucositis. By modulating cytokine profiles and limiting epithelial damage, FVB may offer a valuable strategy for protecting the gastrointestinal tract in cancer patients undergoing chemotherapeutic treatment.

The inflammatory cascade triggered by pro-inflammatory cytokines involves several pathological processes, including increased vascular permeability, recruitment of immune cells to sites of injury, and the amplification of tissue damage. These cytokines also disrupt intestinal homeostasis by compromising tight junction integrity and inducing epithelial cell apoptosis, thereby weakening the intestinal barrier [35]. As a result, the intestinal epithelium becomes more permeable, leading to malabsorption, diarrhea, and heightened susceptibility to microbial translocation and systemic infections. Importantly, the interplay between oxidative stress and cytokine release is bidirectional and synergistic. Oxidative stress, often initiated by chemotherapeutic agents such as 5-FU, stimulates the production of inflammatory cytokines, including TNF-α, IL-1β, and IL-6. These cytokines, in turn, further promote the generation of ROS, establishing a vicious cycle of sustained oxidative and inflammatory damage [36]. For instance, TNF-α has been shown to activate NADPH oxidase, thereby enhancing intracellular ROS levels. This self-perpetuating loop contributes to the chronic inflammation and epithelial damage characteristic of chemotherapy-induced mucositis. The persistent activation of inflammatory and oxidative pathways not only causes significant structural damage to the intestinal mucosa but also impairs its ability to regenerate, delaying the healing process. These effects collectively exacerbate the severity of mucositis and compromise the patient’s overall response to cancer treatment by reducing nutrient absorption and increasing the risk of infection [37]. These results collectively indicate that FVB exerts potent protective effects against 5-FU-induced intestinal injury by significantly attenuating ROS production and suppressing pro-inflammatory cytokine expression in small intestinal tissues. Additionally, FVB inhibited the activation of critical inflammatory signaling pathways, including NF-κB and MAPK cascades, thereby highlighting its therapeutic potential in mitigating chemotherapy-associated mucositis and supporting further translational research. These findings provide critical mechanistic insight into the protective effects of FVB and suggest that it may serve as a promising adjunctive therapy to mitigate chemotherapy-induced mucosal injury. By interrupting the positive feedback loop between oxidative stress and cytokine signaling, FVB may help preserve intestinal integrity and improve patient outcomes during chemotherapy.

As a pivotal component of the innate immune system, TLR4 plays a crucial role in recognizing pathogen-associated molecular patterns (PAMPs) and damage-associated molecular patterns (DAMPs), both of which are indicative of microbial infection or tissue injury [38]. Upon activation, TLR4 initiates downstream signaling cascades that culminate in the activation of transcription factors such as NF-κB, a master regulator of inflammation. A key marker of NF-κB activation is the phosphorylation of its p65 subunit, which facilitates its nuclear translocation and transcriptional activity. The phosphorylation of p65 not only serves as a reliable indicator of TLR4 pathway activation but also highlights the intricate interplay between innate immune recognition and inflammatory signaling, particularly in pathologies such as sepsis, cancer, and chronic inflammatory diseases [39]. In the context of chemotherapy-induced intestinal mucositis, particularly that caused by 5-FU, TLR4/NF-κB signaling is significantly upregulated. This heightened activity leads to the overproduction of pro-inflammatory cytokines [40]. These cytokines are not only central mediators of the inflammatory response, but also major contributors to the epithelial barrier disruption observed in mucositis. Elevated levels of these cytokines in the ileal tissues of 5-FU-treated mice correlate with increased mucosal permeability, epithelial cell apoptosis, and histopathological damage. Our findings demonstrate that activation of the TLR4/NF-κB signaling pathway serves as a key molecular mechanism underlying 5-FU-induced intestinal injury. Moreover, targeting this pathway may offer therapeutic benefits. In our study, treatment with FVB effectively attenuated p65 phosphorylation and reduced the expression of TLR4 and its downstream inflammatory mediators. These results suggest that FVB can modulate TLR4/NF-κB signaling, thereby mitigating cytokine-driven inflammation and preserving intestinal integrity. This highlights the potential of FVB as a protective agent against chemotherapy-induced mucosal toxicity.

The MAPK family is recognized as playing a pivotal role in the pathogenesis of 5-FU-induced intestinal mucositis. As serine/threonine kinases, these signaling molecules are highly responsive to diverse stress stimuli, including oxidative stress, pro-inflammatory cytokines, and chemotherapeutic agents such as 5-FU. Upon activation, MAPKs orchestrate fundamental cellular processes—inflammation, apoptosis, proliferation, and tissue repair—through coordinated downstream signaling [41]. In preclinical models of 5-FU-induced mucositis, elevated MAPK activity has been consistently correlated with exacerbated mucosal inflammation and epithelial injury, implicating these kinases as critical mediators of chemotherapy-associated gastrointestinal toxicity. Beyond amplifying inflammatory cascades, MAPKs also perpetuate the sustained production of inflammatory mediators that aggravate mucosal damage. Notably, the JNK and p38 MAPK subfamilies have been intimately linked to the activation of NF-κB, a master transcriptional regulator of inflammation [42]. These kinases promote NF-κB signaling via phosphorylation of upstream IKK, leading to IκB degradation and NF-κB nuclear translocation. Sustained MAPK activation in response to 5-FU not only drives acute inflammation but also disrupts tissue repair resolution, prolonging mucosal injury. These mechanisms highlight MAPKs as central mediators of 5-FU-induced gastrointestinal toxicity and potential therapeutic targets for intervention [43]. Furthermore, MAPK signaling intersects with apoptotic pathways, exacerbating epithelial cell death and compromising intestinal barrier function. These effects collectively intensify the clinical manifestations of mucositis, such as diarrhea, malabsorption, and weight loss, and may limit the tolerability and effectiveness of chemotherapy. Importantly, interventions that modulate MAPK signaling have shown promise in mitigating the severity of mucositis in preclinical models. In this context, our ongoing investigations explore whether FVB treatment can attenuate the activation of ERK, JNK, and p38 MAPKs following 5-FU exposure, thereby reducing inflammation and promoting mucosal healing [44]. Additionally, MAPKs play an active role in modulating TNF-α expression, a pivotal cytokine in the pathogenesis of mucositis. ROS generation induced by 5-FU therapy was shown to further activate MAPK signaling, which in turn intensifies NF-κB and COX-2 activities, thereby perpetuating a self-reinforcing cycle of oxidative and inflammatory injury [45]. Our results demonstrate that FVB treatment effectively inhibits MAPK phosphorylation in intestinal tissues, while concurrently attenuating NF-κB activation and suppressing the expression of pro-inflammatory cytokines. Collectively, these findings suggest that FVB confers protection against 5-FU-induced mucosal injury through coordinated modulation of the MAPK and NF-κB signaling cascades. This dual anti-inflammatory and antioxidant mechanism underscores its therapeutic potential as a modulator of chemotherapy-associated gastrointestinal toxicity.

Oxidative storm stands at the epicenter of 5-FU-induced mucositis—a ruthless mediator that dismantles epithelial integrity and aggravates inflammatory destruction. As an unavoidable byproduct of its tumoricidal effect, 5-FU unleashes a deluge of ROS that, while devastating to malignant cells, also ravages the rapidly renewing healthy intestinal mucosa. This indiscriminate oxidative assault, collateral to therapeutic intent, serves to potentiate—indeed, to amplify—the very mucosal toxicity that cripples treatment tolerability and patient well-being [46]. Although these ROS effectively target and destroy malignant cells, they simultaneously inflict collateral damage on healthy, rapidly proliferating tissues, particularly the intestinal epithelium. When the endogenous antioxidant defense systems, such as GSH and SOD, become overwhelmed, excessive ROS accumulation triggers oxidative stress, resulting in lipid peroxidation, DNA strand breaks, and protein oxidation. These cellular injuries collectively compromise intestinal barrier integrity and contribute to the development of mucositis [47]. To mitigate oxidative insults, the Nrf2/HO-1 signaling pathway is activated as a protective compensatory response. During oxidative stress, Nrf2 dissociates from its repressor Keap1 and translocates to the nucleus, where it drives the transcription of antioxidant and cytoprotective genes such as HO-1. This mechanism plays a vital role in reducing ROS-induced cellular damage and sustaining mucosal homeostasis under inflammatory conditions [48]. However, sustained or excessive oxidative stress further activates pro-inflammatory signaling pathways, including MAPKs and TLR4/NF-κB. These pathways amplify inflammatory cascades, thereby exacerbating mucosal damage and impeding tissue repair. In this study, we demonstrated that FVB administration significantly attenuated oxidative stress in 5-FU-challenged mice. Mechanistically, FVB enhanced the expression of Nrf2 and its downstream target HO-1, underscoring its role in potentiating the cellular antioxidant defense system. These results collectively demonstrate that FVB treatment attenuates activation of the MAPK and NF-κB signaling pathways, thereby reducing pro-inflammatory cytokine production and improving histopathological features in the small intestine. The findings suggest that FVB alleviates 5-FU-induced mucositis through integrated modulation of oxidative stress and inflammatory responses, primarily by enhancing Nrf2-HO-1 axis activity and suppressing downstream inflammatory cascades. Such evidence underscores FVB’s therapeutic promise as an adjuvant therapy for managing chemotherapy-induced intestinal toxicity, warranting further preclinical and clinical evaluation.

Apoptosis plays a central role in the pathogenesis of 5-FU-induced intestinal mucositis. As a chemotherapeutic agent, 5-FU exerts its antitumor effects primarily by interfering with DNA and RNA synthesis in rapidly proliferating cells, leading to selective cytotoxicity against neoplastic tissue. This mechanism, while effective in targeting malignant cells, also contributes to the induction of apoptosis in normal proliferative epithelial cells of the gastrointestinal mucosa. However, this mechanism is not tumor-specific and also affects normal epithelial cells in the gastrointestinal tract, particularly the highly regenerative crypt stem cells that are essential for maintaining intestinal homeostasis [49]. The loss of these progenitor cells disrupts the equilibrium between epithelial proliferation and differentiation, resulting in villus atrophy, compromised barrier integrity, and increased intestinal permeability. These structural and functional changes lead to impaired nutrient absorption and contribute to the clinical manifestations of mucositis, including diarrhea, weight loss, and malnutrition. These findings highlight the critical role of progenitor cell preservation in maintaining mucosal homeostasis and underscore the potential therapeutic benefit of interventions aimed at protecting these cells during chemotherapy. At the molecular level, 5-FU initiates apoptosis in intestinal epithelial cells via both intrinsic and extrinsic pathways. This process involves an increase in pro-apoptotic proteins such as Bax and cleaved caspase-3, coupled with a decrease in the anti-apoptotic protein Bcl-2. The resulting shift in the Bax/Bcl-2 ratio impairs mitochondrial membrane function, setting off a cascade that includes cytochrome c release and caspase activation, ultimately leading to cell death [50]. This apoptotic response is central to the mucosal toxicity seen with 5-FU, as it compromises the epithelial barrier and delays healing. Therefore, modulating the Bcl-2 family presents a potential therapeutic avenue. In line with this, our study observed that FVB treatment significantly lowered the rate of 5-FU-induced apoptosis in the small intestine [51]. Western blot analysis showed decreased expression of pro-apoptotic proteins Bax and cleaved caspase-3, along with increased levels of the anti-apoptotic protein Bcl-2. These alterations in the apoptotic pathway suggest that FVB exerts a cytoprotective effect by preserving crypt cell viability and maintaining epithelial integrity. Histological analysis further confirmed improved villus height and reduced mucosal inflammation in FVB-treated mice compared to the 5-FU group. These findings highlight FVB’s potential as a protective adjuvant in mitigating chemotherapy-induced intestinal damage. These findings support the hypothesis that FVB alleviates chemotherapy-induced mucositis through the regulation of apoptotic signaling. By restoring the balance between pro- and anti-apoptotic factors, FVB promotes epithelial cell survival and enhances the regenerative capacity of the intestinal mucosa. This protective mechanism may contribute to the observed improvements in clinical outcomes, including reduced weight loss and diarrhea. Collectively, our results highlight the therapeutic potential of FVB as a novel adjunctive strategy for managing 5-FU-induced intestinal toxicity.

Autophagy is a lysosome-mediated degradative pathway essential for the turnover of cytoplasmic constituents—including proteins, lipids, and organelles—thereby playing a fundamental role in the maintenance of cellular homeostasis [50]. It is currently recognized as a tightly regulated process that critically influences cell survival in the context of kidney disease. Furthermore, autophagy functions as a cytoprotective mechanism against nutrient deprivation, aging, and pathogenic invasion in various inflammatory disorders. Under conditions of metabolic stress, autophagy is activated to eliminate damaged organelles, sustain ATP generation, and support protein synthesis [51]. However, excessive oxidative stress can provoke cellular injury and disturb the expression of autophagy-related markers, such as LC3-II/I, Beclin-1, and p62. In the present study, FVB treatment significantly downregulated the protein levels of LC3-II, p62, and Beclin-1, suggesting a link between autophagic dysregulation and 5-FU-induced intestinal toxicity in mice.

5-FU-induced intestinal mucositis is characterized by widespread epithelial injury along the gastrointestinal tract, culminating in chronic inflammation, disruption of barrier integrity, and defective tissue regeneration. A central pathological mechanism underlying this condition is EMT, a cellular reprogramming event in which epithelial cells progressively lose their apical–basal polarity and intercellular tight junctions, concurrently acquiring a mesenchymal phenotype defined by increased migratory capacity and invasive potential. In the context of 5-FU-induced mucositis, the downregulation of epithelial markers—including E-cadherin and β-catenin—compromises adherens junction stability, thereby perturbing epithelial cohesion and precipitating barrier dysfunction [52]. This increased permeability heightens the vulnerability of the intestinal lining to pathogen invasion and luminal antigen exposure, which further amplifies the inflammatory response. Simultaneously, the upregulation of mesenchymal markers such as N-cadherin reflects a phenotypic shift that reinforces tissue remodeling and damage, exacerbating the severity of mucosal injury. The role of EMT in mucositis is further compounded by a feedforward inflammatory loop. Pro-inflammatory cytokines can induce EMT, compromising epithelial integrity and enhancing mucosal permeability [53]. In turn, this barrier dysfunction promotes the release of additional inflammatory mediators, which sustain and escalate the inflammatory environment. This bidirectional interaction not only accelerates tissue damage but also delays epithelial restitution, contributing to the chronicity of mucosal lesions observed during chemotherapy. Interestingly, EMT also plays a dual role in the intestinal epithelium. While excessive or prolonged EMT contributes to inflammation and damage, controlled EMT may be involved in wound healing and epithelial regeneration. Therefore, therapeutic strategies aimed at modulating EMT-related signaling pathways must strike a balance between mitigating injury and preserving regenerative capacity [54]. In our study, treatment with FVB significantly attenuated 5-FU-induced EMT in murine models. Western blot analysis revealed that FVB administration upregulated β-catenin and E-cadherin expression, thereby reinforcing epithelial phenotype and intercellular adhesion. Concurrently, FVB reduced N-cadherin levels, suggesting suppression of mesenchymal transformation. These molecular changes correlated with histological improvements, including restored villus architecture and reduced mucosal inflammation. Collectively, these findings suggest that FVB alleviates 5-FU-induced mucositis by regulating EMT-associated pathways. By preserving epithelial identity and preventing the transition to a mesenchymal phenotype, FVB strengthens barrier integrity, reduces inflammatory signaling, and promotes mucosal healing. These results highlight the EMT pathway as a viable therapeutic target and underscore FVB’s potential as an adjunctive agent in managing chemotherapy-induced gastrointestinal complications.

Tight junctions—the sentinels of intestinal epithelial sovereignty—are systematically dismantled in 5-FU-induced mucositis, as replicated in preclinical murine models. These multicomponent gatekeepers, forged from transmembrane occludin and claudins and anchored by cytoplasmic ZO-1 scaffolds, rigidly patrol paracellular chokepoints. In so doing, they protect against a hostile luminal menagerie—pathogens, toxins, antigens—barring its ingress into the sterile subepithelial realm. Their breach heralds barrier capitulation, unleashing a torrent of inflammatory insult that defines mucositis pathology [55]. Following 5-FU treatment, a significant reduction in ZO-1, a key scaffolding protein that tethers tight junctions to the actin cytoskeleton, was observed. This change compromised tight junction structural integrity and increased intestinal permeability. Similarly, the expression of occludin, a transmembrane protein crucial for tight junction function, was substantially decreased after 5-FU exposure, impairing epithelial barrier integrity. These alterations highlight the vulnerability of tight junction proteins to chemotherapy-induced damage and underscore their importance as potential therapeutic targets for mitigating mucosal toxicity [56]. Claudin-1, a key transmembrane tight junction protein, exhibited significant downregulation after 5-FU treatment, leading to increased paracellular permeability and an influx of inflammatory mediators that exacerbate mucosal injury. The reduction in tight junction proteins (ZO-1, occludin, claudin-1) reflects 5-FU toxicity, mediated by oxidative stress and ROS overproduction, which disrupts tight junction protein expression [57]. TNF-α and IL-6—potent inflammatory instigators—actively dismantle tight junction fortifications. Our investigation establishes that FVB decisively shields occludin, claudins, and ZO-1 from 5-FU-inflicted erosion, preserving the epithelial barricade. By reinstating the molecular rivets that seal the paracellular route, FVB emerges as a compelling therapeutic sentinel—uniquely poised to restore barrier fidelity and quell the pathogenesis of 5-FU-induced mucositis.

The principal objective of the present investigation was to evaluate the capacity of FVB to confer protection against 5-FU-induced intestinal injury; consequently, a cohort receiving FVB alone was not incorporated into the experimental design. Nevertheless, multiple lines of evidence substantiate the safety of FVB at the administered dosage. First, no overt signs of systemic toxicity or alterations in behavior were evident in animals receiving FVB. Second, FVB is derived from an edible fungus with a long-established history of safe human consumption. Third, prior studies have demonstrated that FVB ameliorates cisplatin-induced acute and chronic kidney injury without discernible adverse effects [13,14]. Furthermore, in a dedicated 14-day safety assessment conducted in our laboratory, oral administration of FVB at 1.0 g/kg resulted in no significant deviation in body weight, and histopathological evaluation (H&E staining) of hepatic, renal, and pulmonary tissues revealed no morphological abnormalities. These findings collectively confirm the safety profile of FVB under the experimental conditions employed in this study.

FVB is known to harbor a rich repertoire of polyphenolic compounds—bioactive phytochemicals celebrated for their potent pharmacological properties. The hydroxyl (–OH) moieties decorating these polyphenolic architectures act as proficient hydrogen donors, efficiently quenching reactive oxygen species (ROS) and thus mounting a formidable antioxidant defense [58]. In addition to their antioxidative capacity, these polyphenols possess anti-inflammatory and anticancer properties through modulation of multiple cellular signaling pathways, including NF-κB, MAPKs, and apoptotic regulators. In our previous study, HPLC analyses confirmed that FVB extracts contain substantial quantities of bioactive polyphenols, supporting their potential as a source of natural bioactive agents for therapeutic applications in oxidative stress-related pathologies, including chemotherapy-induced mucositis. Specifically, comprehensive phytochemical profiling led to the identification and quantification of two major phenolic acid constituents, with particular emphasis on quercetin and gallic acid [59]. These two compounds have been extensively studied for their broad-spectrum therapeutic properties, including free radical scavenging, anti-inflammatory effects, and their ability to modulate gene expression related to oxidative stress and cell survival [15].

The natural flavonoid quercetin is widely distributed in the plant kingdom. It is abundantly found in numerous common foods, ranging from tea, apples, and red wine to onions, broccoli, kale, oranges, and blueberries, among others. Its most prominent characteristic lies in its potent antioxidant activity; however, its oral bioavailability remains notably low. Quercetin exerts protective effects against cellular oxidative damage through multiple mechanisms, such as scavenging ROS, inhibiting lipid peroxidation, suppressing xanthine oxidase activity, and chelating metal ions. Additionally, it demonstrates antiallergic, anti-inflammatory, and antiviral properties [59]. Quercetin has been shown to inhibit tumor progression in various malignancies, including prostate, liver, lung, breast, colon, and cervical cancers. Notably, quercetin undergoes biotransformation by tyrosinase into active metabolites with enhanced anticancer potential. Recent findings suggest that co-administration of quercetin with cisplatin yields synergistic anticancer effects. In experimental models, quercetin has shown promise in attenuating chemotherapeutic agent-induced intestinal mucositis, such as that caused by 5-FU, by suppressing ROS formation and modulating downstream inflammatory pathways, particularly via downregulation of NF-κB and HIF-1α [60]. Gallic acid, a widely distributed natural polyphenol, is found in sources such as Cornus officinalis, pomegranate, gallnut, peony bark, and tea. Its molecular structure includes multiple hydrogen bond donors and acceptors, facilitating its interaction with biological targets. Gallic acid possesses diverse pharmacological properties, including antioxidant, anti-inflammatory, antiviral, antitumor, and hypoglycemic activities. As an FDA-designated GRAS (Generally Recognized As Safe) compound, gallic acid is well-tolerated and capable of synergizing with chemotherapeutic agents. Reports in the literature indicate that the combination of 5-FU and gallic acid results in synergistic antitumor effects following eutectic dissolution [61].

These results suggest that certain natural compounds found in FVB could help protect the intestines from the damage caused by 5-FU chemotherapy. They seem to work in several ways: by fighting harmful oxidative stress, reducing inflammation, preventing cell death, and helping the gut lining to heal. These combined effects may help reduce side effects, limit damage to healthy tissue, and possibly allow lower doses of chemotherapy to be used. Researchers also discovered that certain chemical structures in these compounds, such as the catechol group and a special type of carbon bond, play key roles in how they work. Still, more research is needed to understand exactly how these compounds interact with the body on a molecular level.

## 4. Materials and Methods

### 4.1. Preparation of Samples

The FVB sample utilized in the present study was cultivated under controlled environmental conditions at Wanshen Mushroom Farm (Nantou, Taiwan). To ensure extract quality and minimize substrate contamination, the basal 2 cm portion of each fruiting body was excised prior to extraction. The remaining tissue was subjected to distilled water extraction at a 1:4 (*w*/*w*) ratio using an autoclave-based procedure (121 °C, 1.5 kg/cm^2^, 15 min) repeated twice. The resulting crude extract was filtered through a cloth press to remove insoluble particulates, and the filtrate was subsequently concentrated under vacuum at 50 °C employing a water bath system (PANCHUM, Kaohsiung, Taiwan). The concentrated extract was then lyophilized (Kingmech, New Taipei, Taiwan) to obtain a freeze-dried powder, which was stored at −20 °C until further use. The overall extraction yield was determined to be 3.21% (*w*/*w*). Based on preliminary toxicity and efficacy evaluations, oral doses of 0.5 g/kg and 1.0 g/kg body weight were selected for subsequent animal experiments.

### 4.2. Reagents

Reagents used in this study included 5-FU, mesalazine, and various solvents from Sigma-Aldrich (St. Louis, MO, USA). For immunoblotting analyses, primary antibodies against a broad spectrum of inflammatory and apoptotic markers were applied at a 1:1000 dilution, including iNOS, COX-2, TLR4, NF-κB, p-NF-κB, PI3K, p-PI3K, AKT, p-AKT, p-ERK, ERK, p-JNK, JNK, p-p38, p38, Bax, Bcl-2, and caspase-3 (Cell Signaling Technology, Danvers, MA, USA). Antibodies for antioxidant enzymes (catalase, SOD1, GPx3), junction proteins (β-catenin, E-cadherin, N-cadherin, occludin, claudin-1, ZO-1), and redox regulators (HO-1, Nrf2) were acquired from Abcam and used at optimal dilutions (1:1000–1:2000). β-actin (1:10,000) was used as the loading control in all immunoblotting assays.

### 4.3. Animals

Male BALB/c mice (8 weeks of age; mean body weight 25 ± 3 g) were acquired from BioLASCO Taiwan Co., Ltd. (Taipei, Taiwan), a certified commercial vendor. Upon receipt, the animals were immediately transferred to a climate-controlled vivarium operating under an invariant 12:12 h light/dark photoperiod, with ambient temperature and relative humidity meticulously maintained at 23 ± 1 °C and 50 ± 10%, respectively. A mandatory 3- to 5-day acclimatization period was rigorously enforced before any experimental intervention. All procedures described herein were reviewed and formally sanctioned by the Institutional Animal Care and Use Committee of China Medical University (IACUC Approval No. CMUI-ACUC-2025-012), ensuring full compliance with established ethical guidelines.

### 4.4. Research Design

A total of 25 male BALB/c mice were enrolled in this study and randomly allocated into five experimental groups (n = 5 per group): (1) a control group receiving intraperitoneal (i.p.) saline; (2) a 5-fluorouracil (5-FU) group receiving 50 mg/kg 5-FU (i.p.); (3) a mesalazine group receiving 10 mg/kg mesalazine (i.p.) plus 50 mg/kg 5-FU (i.p.); (4) a low-dose FVB group receiving 0.5 g/kg FVB oral suspension plus 5-FU (i.p.); and (5) a high-dose FVB group receiving 1.0 g/kg FVB oral suspension plus 5-FU (i.p.). From day 1, mice in the control and 5-FU groups received daily oral gavage of physiological saline for 10 consecutive days, whereas those in the remaining groups received their respective treatments over the same period. All groups except the control were administered intraperitoneal 5-FU (50 mg/kg) from day 4 to day 7. On day 10, mice were subjected to a 16 h fasting period before anesthesia with pentobarbital and subsequent blood collection. Serum samples were preserved at −20 °C for subsequent biomarker analyses. Small intestinal tissues were immediately harvested for histopathological evaluation and Western blot analysis of a comprehensive panel of proteins, including iNOS, COX-2, TLR4, PI3K, p-PI3K, AKT, p-AKT, NF-κB, p-NF-κB, p-ERK, ERK, p-JNK, JNK, p-p38, p38, Bax, Bcl-2, caspase-3, catalase, SOD, GPx3, β-catenin, E-cadherin, N-cadherin, occludin, claudin-1, ZO-1, HO-1, and Nrf2. Body weight and diarrhea severity were monitored daily throughout the experimental period. Histopathological examinations were performed on all collected specimens (Figure 1A). This experimental design enabled a systematic evaluation of the protective effects of FVB against 5-FU-induced intestinal mucositis [62].

### 4.5. Daily Weight and Diarrhea Monitoring

Mouse body weight and diarrhea scores were recorded daily. Two independent observers confirmed each score, and averages were calculated. Diarrhea was graded on a 0–4 scale: 0 = normal; 1 = soft; 2 = slightly wet; 3 = unformed with moderate staining; and 4 = watery with severe staining. Severity was assessed by incidence and mean score [7].

### 4.6. Histological Examination

Histopathological evaluation was performed on 5 μm paraffin-embedded intestinal tissue sections stained with hematoxylin and eosin (H&E) and subsequently examined under a Nikon Eclipse TS100 light microscope (Nikon Corporation, Tokyo, Japan). The severity of intestinal injury was quantitatively assessed using a standardized grading scale ranging from 0 to 4, with grade 4 defined as involvement of more than 75% of the tissue area. To evaluate epithelial regeneration during the recovery phase, immunohistochemical staining for Ki-67 was conducted. Briefly, tissue sections were deparaffinized, subjected to antigen retrieval, blocked to minimize nonspecific binding, and incubated with a primary antibody against Ki-67 at 4 °C for 24 h. Immunoreactivity was visualized using diaminobenzidine (DAB) chromogen (Sigma-Aldrich, St. Louis, MO, USA).

### 4.7. Cytokine Assay

The levels of the canonical pro-inflammatory effectors—TNF-α, IL-1β, and IL-6—were interrogated by a high-sensitivity enzyme-linked immunosorbent assay (ELISA) employing validated kits sourced from BioLegend (San Diego, CA, USA). Adherence to the manufacturer’s stipulated protocols was rigorously enforced throughout, thereby safeguarding the veracity and technical robustness of the resultant quantitative readouts.

### 4.8. Nitrite Assay

Nitrite concentration in culture supernatants was determined using the Griess colorimetric assay. Briefly, 100 μL of Griess reagent was added to each sample, followed by incubation at ambient temperature for 10 min. Absorbance was measured at 540 nm using a microplate reader (Molecular Devices, Sunnyvale, CA, USA). Nitrite levels were subsequently calculated and employed as a surrogate marker for NO production [63].

### 4.9. The TBARS (Thiobarbituric Acid-Reactive Substance) Assay

Malondialdehyde (MDA)—a well-established biomarker of thiobarbituric acid-reactive substances (TBARSs) and lipid peroxidation—was quantified in small intestinal tissue homogenates. Samples were homogenized in ice-cold lysis buffer and subsequently incubated with thiobarbituric acid (TBA) solution at 90 °C for 45 min. The formation of MDA–TBA adducts was then measured spectrophotometrically at 532 nm, providing a reliable index of oxidative lipid damage [64].

### 4.10. Glutathione (GSH) Assay

The prototypic sulfhydryl-detecting agent Ellman’s reagent (5,5′-dithiobis (2-nitrobenzoic acid); DTNB) was deployed as the chromogenic cornerstone for glutathione (GSH) quantitation. A precisely formulated reaction mixture was prepared: 100 μL of clarified supernatant, 200 μL of 0.3 M phosphate buffer (pH 8.4), 400 μL of double-distilled water, and 500 μL of DTNB chromophore. Chromogenic development was investigated spectrophotometrically at 412 nm, capturing the stoichiometric release of 5-thio-2-nitrobenzoate. Parallel assessment of total protein content was executed via the Bradford dye-binding methodology (Bio-Rad Laboratories, Hemel Hempstead, UK), providing an essential normalization parameter for the derived GSH values [64].

### 4.11. Western Blot Analysis

Total protein lysates were harvested from small intestinal explants via mechanical disruption in RIPA buffer thoroughly supplemented with a broad-spectrum protease inhibitor cocktail. The solubilized proteome was resolved by denaturing SDS-PAGE and subsequently electro-immobilized onto high-binding-capacity PVDF or nitrocellulose membranes. These protein-laden matrices were then subjected to a meticulously choreographed immunodetection cascade: incubation with primary antibodies of predefined specificity, followed by stringent washing and exposure to species-matched HRP-conjugated secondary antibodies (anti-rabbit or anti-mouse IgG). The antigen–antibody complexes were rendered visible through enzyme-catalyzed enhanced chemiluminescence (ECL), and the resultant chemiluminescent fingerprints were digitally archived and quantitatively deciphered using Kodak Molecular Imaging Software 5.0 (Eastman Kodak Company, Rochester, NY, USA).

### 4.12. Statistical Analysis

All quantitative data are expressed as mean ± standard deviation (S.D.). Statistical comparisons between two groups were performed using an unpaired two-tailed Student’s *t*-test. For comparisons involving three or more groups, one-way analysis of variance (ANOVA) was employed, followed by Scheffé’s post hoc test for multiple comparisons. A *p*-value of less than 0.05 was considered to indicate statistical significance.

## 5. Conclusions

FVB demonstrated significant therapeutic promise in mitigating 5-FU-induced intestinal mucositis by modulating oxidative stress and reducing associated mucosal damage. Through its potent antioxidant and anti-inflammatory mechanisms, FVB effectively suppressed major pathological events, including apoptosis, inflammation, EMT, and tight junction disruption. While these preclinical findings are highly encouraging, further studies are essential to validate and expand FVB’s therapeutic applications to other inflammatory and oxidative stress-related disorders. Therefore, FVB emerges as a promising adjuvant to counteract chemotherapy-induced intestinal toxicity and protect mucosal integrity.

## Data Availability

The original contributions presented in this study are included in the article. Further inquiries can be directed to the corresponding authors.
